# Data of The Expression of Serotonin in Alzheimer's Disease (AD) Rat Model Under Treatment of Ethanolic Extract Ocimum sanctum Linn

**DOI:** 10.1016/j.dib.2020.105654

**Published:** 2020-04-30

**Authors:** M. Nova Raditya, A.M. Made Bagus, Ulayatul Kustiati, Hevi Wihadmadyatami, Dwi Liliek Kusindarta

**Affiliations:** a Department of Anatomy, Faculty of Veterinary Medicine, Universitas Gadjah Mada, Yogyakarta, 55281, Indonesia; b Integrated Laboratory for Research and Testing, Universitas Gadjah Mada, Yogyakarta, 55281, Indonesia

**Keywords:** Neurodegenerative disease, Hippocampal, Serotonin, *Ocimum sanctum* Linn. Ethanolic extract

## Abstract

The article offers dataset on the expression of serotonergic nerve in the hippocampal area of Alzheimer's disease (AD) model. Since decreasing expression of serotonin linked to dementia, this data will help the neuroscientist, who work on neurodegeneration. This dataset demonstrates the potential of *Ocimum sanctum* Extract (OSE) as a neuroprotective and neurodegenerative agent against AD. The OSE mechanism focusing on the expression of serotonin as a therapeutic target. To acquire the dataset, we approached using two models, in vitro and in vivo. On the In vivo model, used two months old 27 male rats and divided into three groups, non-treated (Group A, n=9), AD rats model pre-treated with OSE followed induction for TMT on the days of seventh (group B, n=9) and AD rats model treated with OSE both on pre-TMT introduction for seven days and post-TMT induction for 21 days (group C, n=9). AD rats euthanised on day seventh; 14th; and 21st. The brain samples were analysed for neuronal density and serotonin immunoreactivity qualitatively. Besides, In Vitro's data were collected from HEK-293 cells which induce by TMT as of AD model. The data expression of serotonin on the in-vitro model analysed using ELISA method.

Specifications Table

**Table:** 

Subject	Ageing
Specific subject area	Neuroscience, herbal medicine, neuropeptide
Type of data	Table Image Chart
How data were acquired	In vivo Model (AD Rat Model), In vitro model (HEK-293 cell line), Immunology technic (immunohistochemistry and ELISA), Microscope, microtechnique (Cresyl violet staining), Analysis by Graph Pad Prism 7 and Optilab.
Data format	Images Graph/ Table Raw data
Parameters for data collection	The rat model AD divided become three groups, after treatment the in vivo model then euthanized. The brain sample derived from the rat model stained with cresyl violet to visualize the Nissl bodies, furthermore to derived the expression of serotonin, the immunohistochemistry applied by using the antibodies against serotonin. For the in vitro model (HEK-293 cells), the data were collected from the lysate of the cells.
Description of data collection	The analysis on the in vivo model was done by image raster software for the quantitative data (supplementary data) and semi-quantitatively data (scoring or grading). Furthermore, for the in-vitro model AD (HEK-293) the expression of serotonin on the cells lysate measured using a spectrophotometer, then the data analysed with GraphPad Prism7 software.
Data source location	Institution: Department of Anatomy, Faculty of Veterinary Medicine, Universitas Gadjah Mada City/Town/Region: Yogyakarta/ Special Region of Yogyakarta Country: Indonesia
Data accessibility	Within the article and in raw supplementary material as mendeley data set, DOI:10.17632/4g74bxfvwr.1 (https://data.mendeley.com/datasets/4g74bxfvwr/1)
Related research article	Kusindarta DL, Wihadmadyatami H, Jadi AR, Karnati S, Lochnit G, Hening P, Haryanto A, Auriva MB, Purwaningrum M. Ethanolic extract Ocimum sanctum. Enhances cognitive ability from young adulthood to middle aged mediated by increasing choline acetyl transferase activity in rat model. Research in Veterinary Science, doi:10.1016/j.rvsc.2018.04.005

## Value of the data

• This dataset revealed the ability of *Ocimum sanctum* ethanolic extract (OSE), one of traditional herbal as a medication against the signs of neurodegenerative diseases.
• This dataset will develop new insight into herbal product thus may bring benefit to the community, scientist, physician, especially who involve on the neurodegenerative disease (NDD) mainly on the Alzheimer's disease (AD) management and prevention.
• These data provide an approach to the level of molecular mechanisms that brings us to initiate more deeply into the AD whole mechanism.

## 1 Data Description

**Fig. 1:**
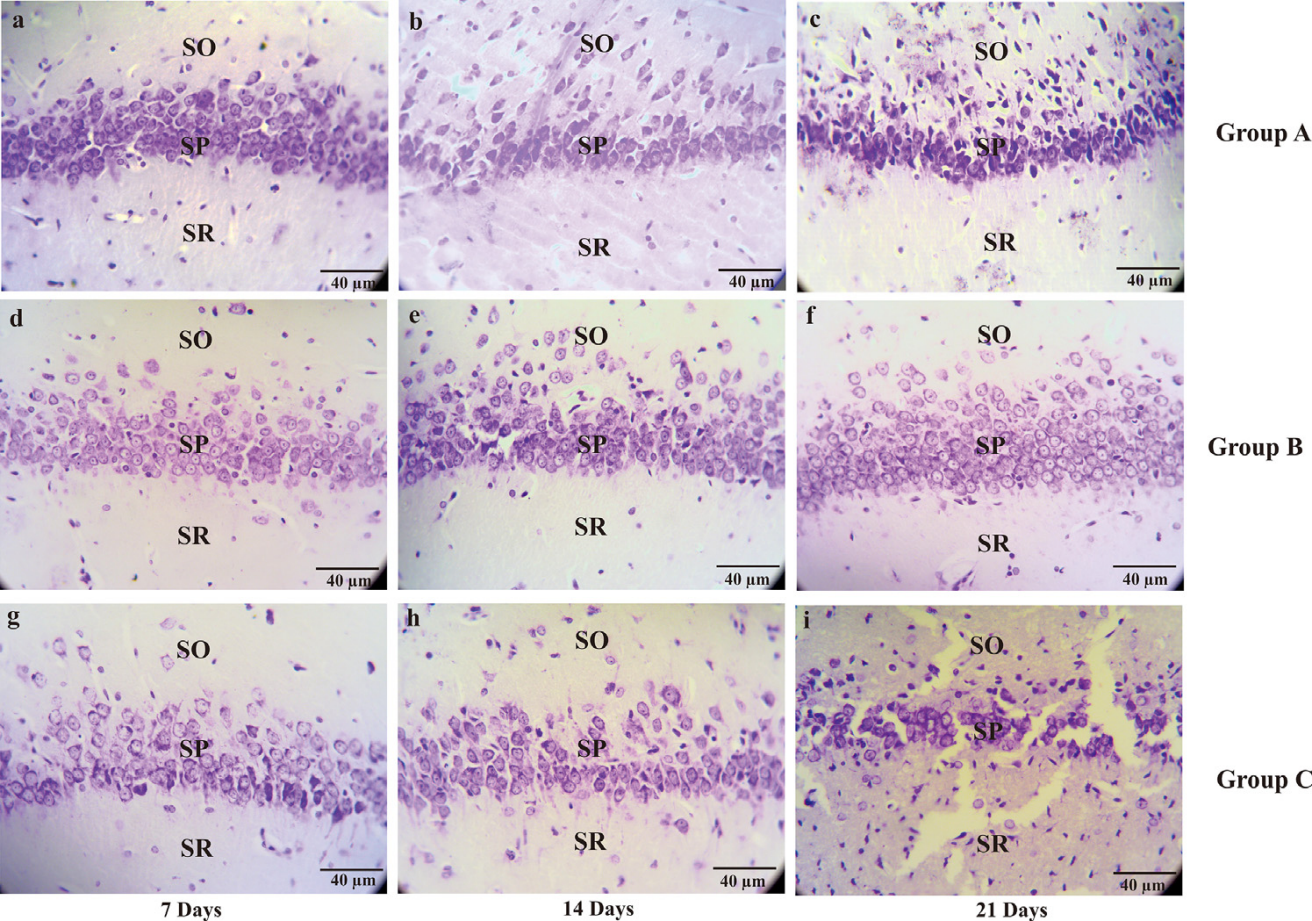
The micrograph represents the neuronal density of CA1 hippocampal in the Alzheimer's disease (AD) rat model. Pyramidal cells on the CA1 hippocampal at the days of seventh, 14^th^, 21^st^ were stain by cresyl violet. Non-treated group (group A) sacrificed on day 7^th^ (A); day 14^th^ (B); day 21^st^ (C), AD rats model (group B) treated with OSE pre-TMT induction for seven days then sacrificed on day seventh (D); day 14^th^ (E); day 21^st^ (F) and AD rats model (group C) treated with OSE pre-TMT induction for seven days and post-TMT induction for 21 days then sacrificed on day seventh (G); day 14^th^ (H); and day 21^st^ (I). SO = Stratum Oriens; SP = Stratum Pyramidale; SR = Stratum Radiatum (40x magnifications, scale bar 40 µm).

**Fig. 2:**
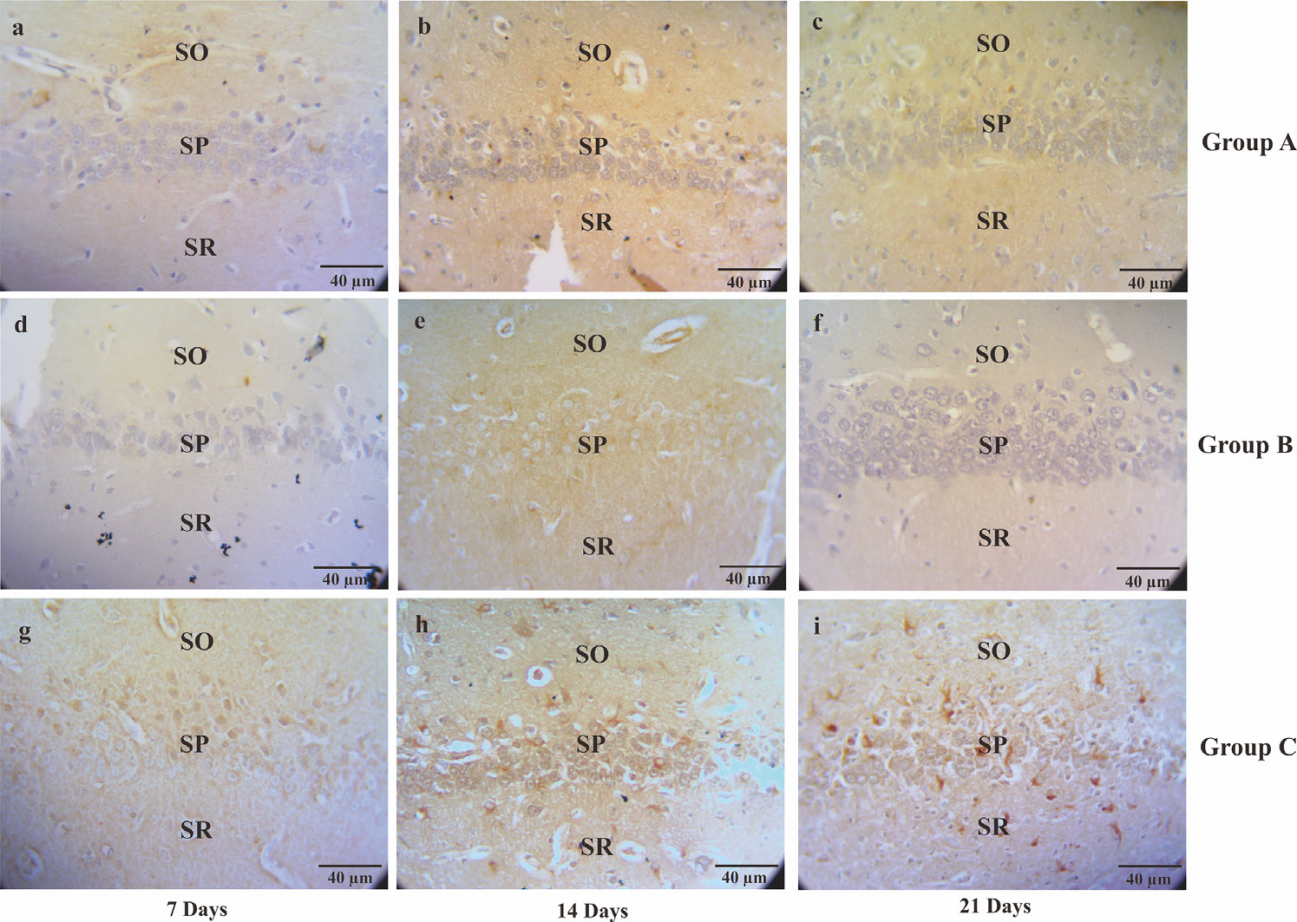
The micrograph describes the serotonin immunoreactivity cells of CA1 hippocampal in the Alzheimer's disease (AD) rat model. The serotonin immunoreactivity cells visualized by Streptavidin-based immunohistochemistry method. Non-treated group (group A) sacrificed on day seventh (A); day 14^th^ (B); day 21^st^ (C), AD rats model (group B) treated with OSE pre-TMT induction for seven days then sacrificed on day seventh (D); day 14^th^ (E); day 21^st^ (F) and AD rats model (group C) treated with OSE pre-TMT induction for seven days and post-TMT induction for 21 days then sacrificed on day seventh (G); day 14^th^ (H); and day 21^st^ (I). SO = Stratum Oriens; SP = Stratum Pyramidale; SR = Stratum Radiatum (40x magnifications, scale bar 40 µm).

**Fig. 3:**
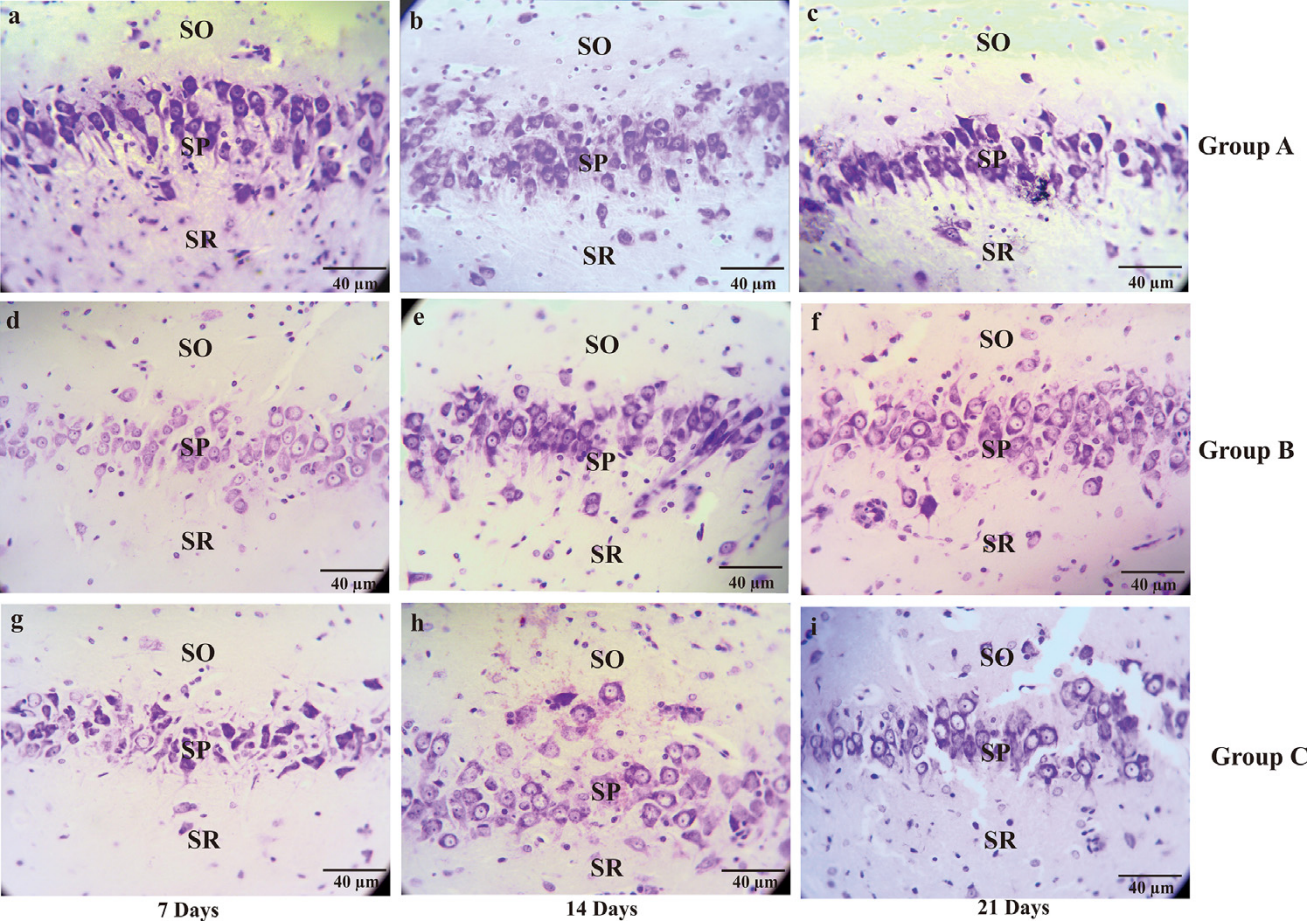
The microscopic picture represents the neuronal density of CA3 hippocampal in the Alzheimer's disease (AD) rat model. Pyramidal cells on the CA3 hippocampal at the days of seventh, 14^th^, 21^st^ stained by cresyl violet. Non-treated group (group A) sacrificed on day seventh (A); day 14^th^ (B); day 21^st^ (C), AD rats model (group B) treated with OSE pre-TMT induction for seven days then sacrificed on day seventh (D); day 14^th^ (E); day 21^st^ (F) and AD rats model (group C) treated with OSE pre-TMT induction for seven days and post-TMT induction for 21 days then sacrificed on day seventh (G); day 14^th^ (H); and day 21^st^ (I). SO = Stratum Oriens; SP = Stratum Pyramidale; SR = Stratum Radiatum (40x magnifications, scale bar 40 µm).

**Fig. 4:**
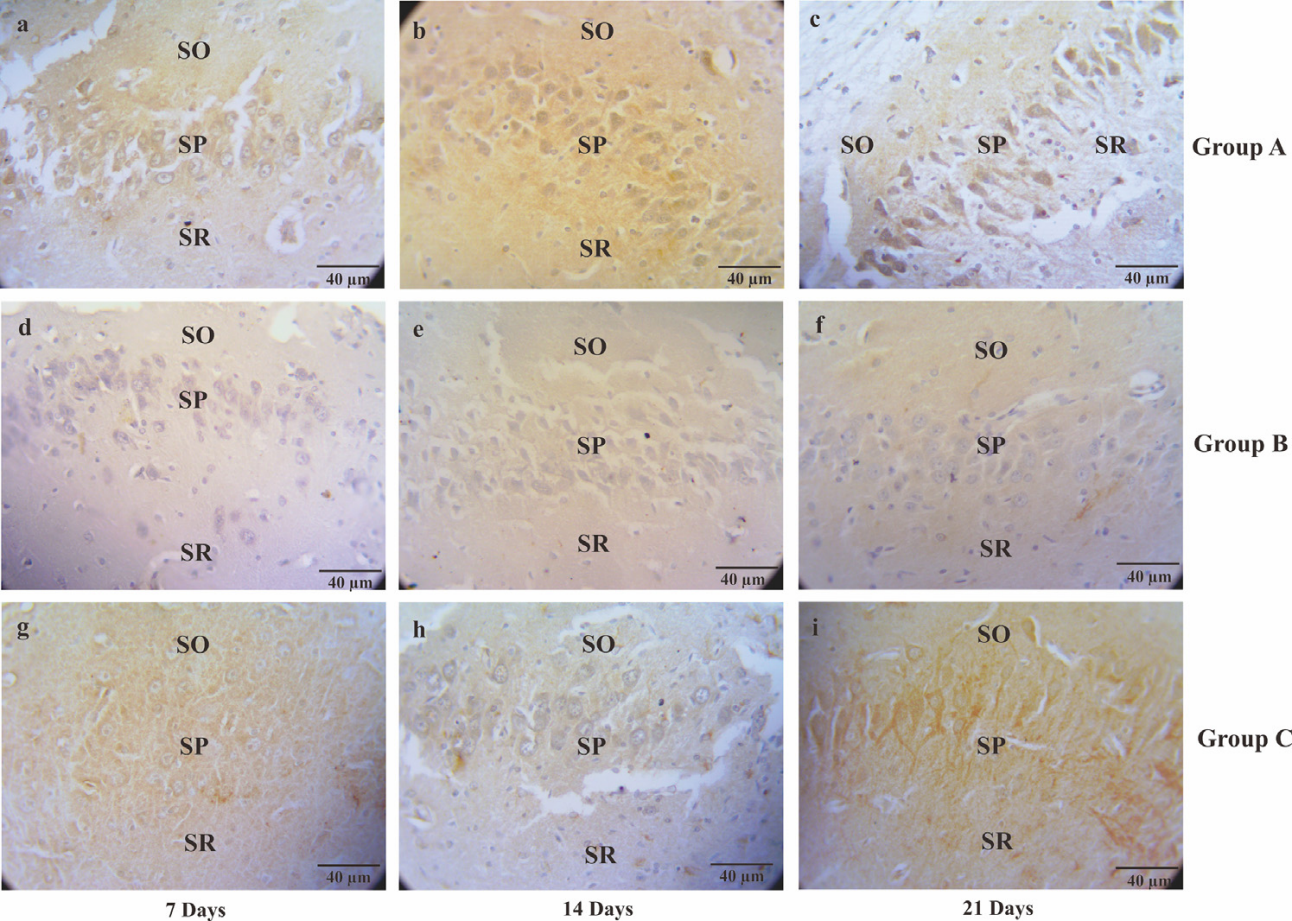
The micrograph describes the serotonin immunoreactivity cells of CA3 hippocampal in the Alzheimer's disease (AD) rat model. The serotonin immunoreactivity cells visualized by Streptavidin-based immunohistochemistry method. Non-treated group (group A) sacrificed on day seventh (A); day 14^th^ (B); day 21^st^ (C), AD rats model (group B) treated with OSE pre-TMT induction for seven days then sacrificed on day seventh (D); day 14^th^ (E); day 21^st^ (F) and AD rats model (group C) treated with OSE pre-TMT induction for seven days and post-TMT induction for 21 days then sacrificed on day seventh (G); day 14^th^ (H); and day 21^st^ (I). SO = Stratum Oriens; SP = Stratum Pyramidale; SR = Stratum Radiatum (40x magnifications, scale bar 40 µm).

**Fig. 5:**
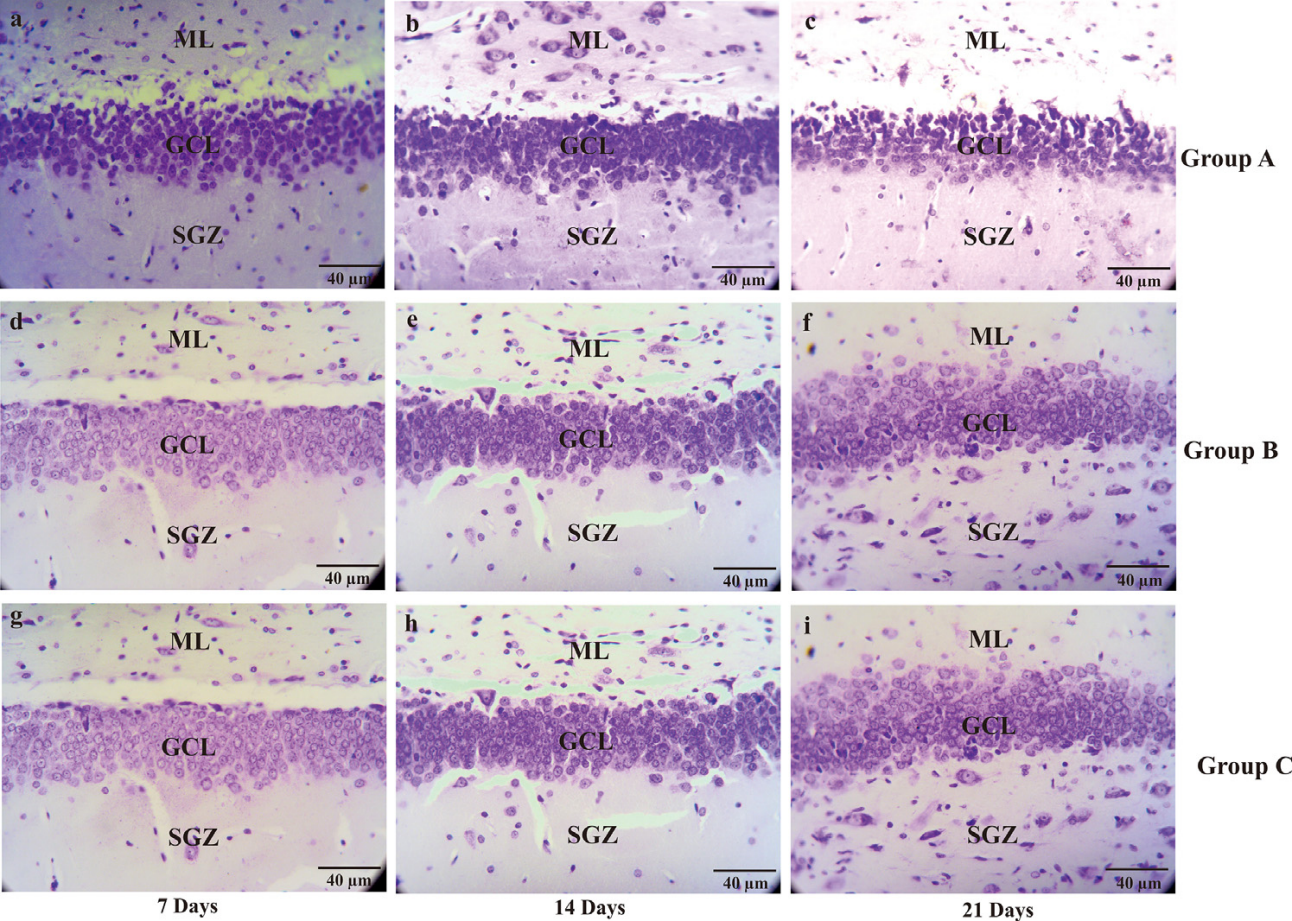
The micrograph represents the neuronal density of DG hippocampal in the Alzheimer's disease (AD) rat model. Granular cells on the DG hippocampal at the days of seventh, 14^th^, 21^st^ were stain by cresyl violet. Non-treated group (group A) sacrificed on day seventh (A); day 14^th^ (B); day 21^st^ (C), AD rats model (group B) treated with OSE pre-TMT induction for 7 days then sacrificed on day seventh (D); day 14^th^ (E); day 21^st^ (F) and AD rats model (group C) treated with OSE pre-TMT induction for seven days and post-TMT induction for 21 days then sacrificed on day seventh (G); day 14^th^ (H); and day 21^st^ (I). ML = Molecular Layer; GCL = Granule Cell Layer; SGZ = Sub-granular zone (40x magnifications, scale bar 40 µm).

**Fig. 6:**
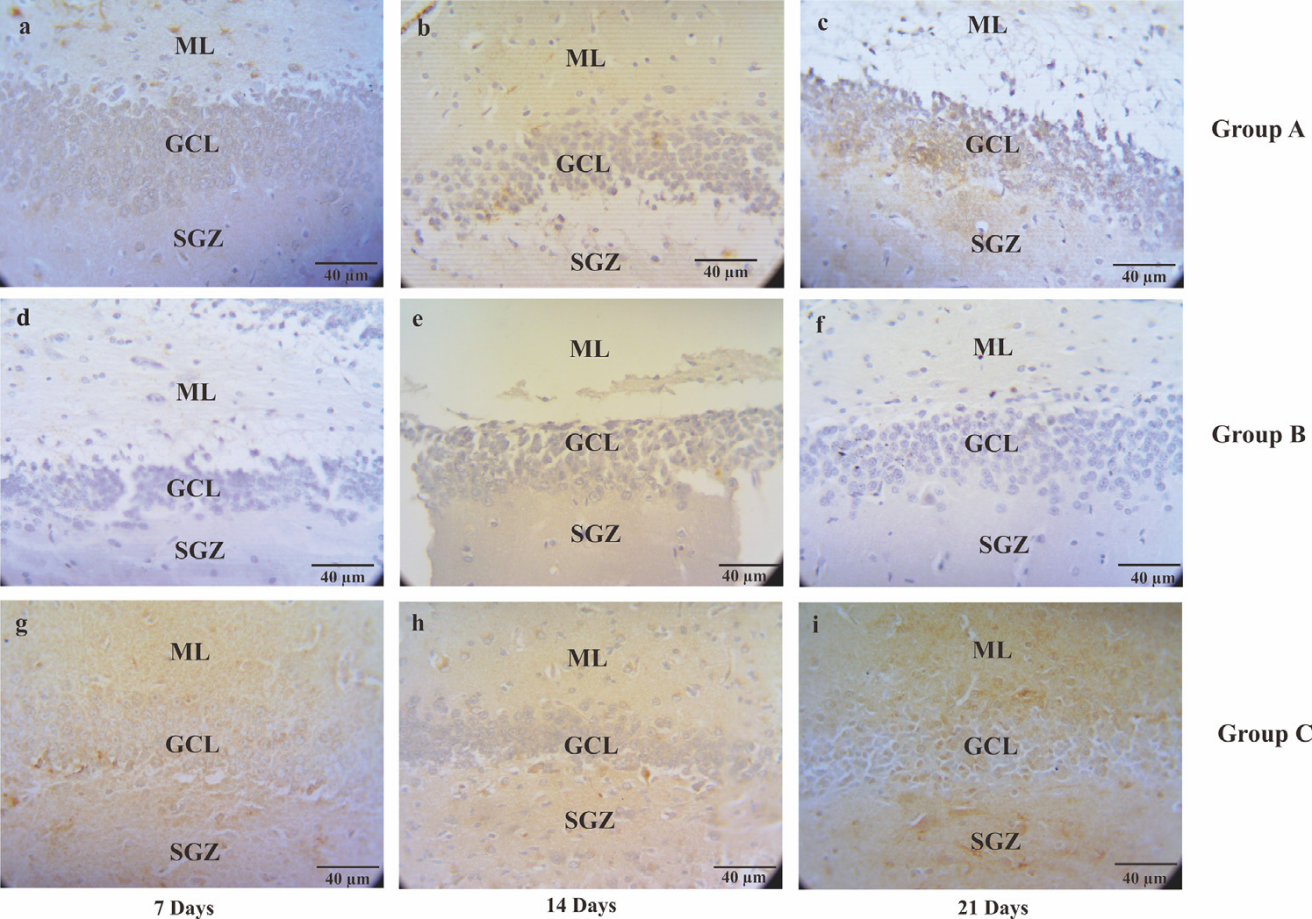
The micrograph picture describes the serotonin immunoreactivity cells of DG hippocampal in the Alzheimer's disease (AD) rat model. The serotonin immunoreactivity cells visualized using Streptavidin-based immunohistochemistry method. Non-treated group (group A) sacrificed on day seventh (A); day 14^th^ (B); day 21^st^ (C), AD rats model (group B) treated with OSE pre-TMT induction for seven days then sacrificed on day seventh (D); day 14^th^ (E); day 21^st^ (F) and AD rats model (group C) treated with OSE pre-TMT induction for seven days and post-TMT induction for 21 days then sacrificed on day seven (G); day 14^th^ (H); and day 21^st^ (I). ML = Molecular Layer; GCL = Granule Cell Layer; SGZ = Sub-granular zone (40x magnifications, scale bar 40 µm).

**Fig. 7:**
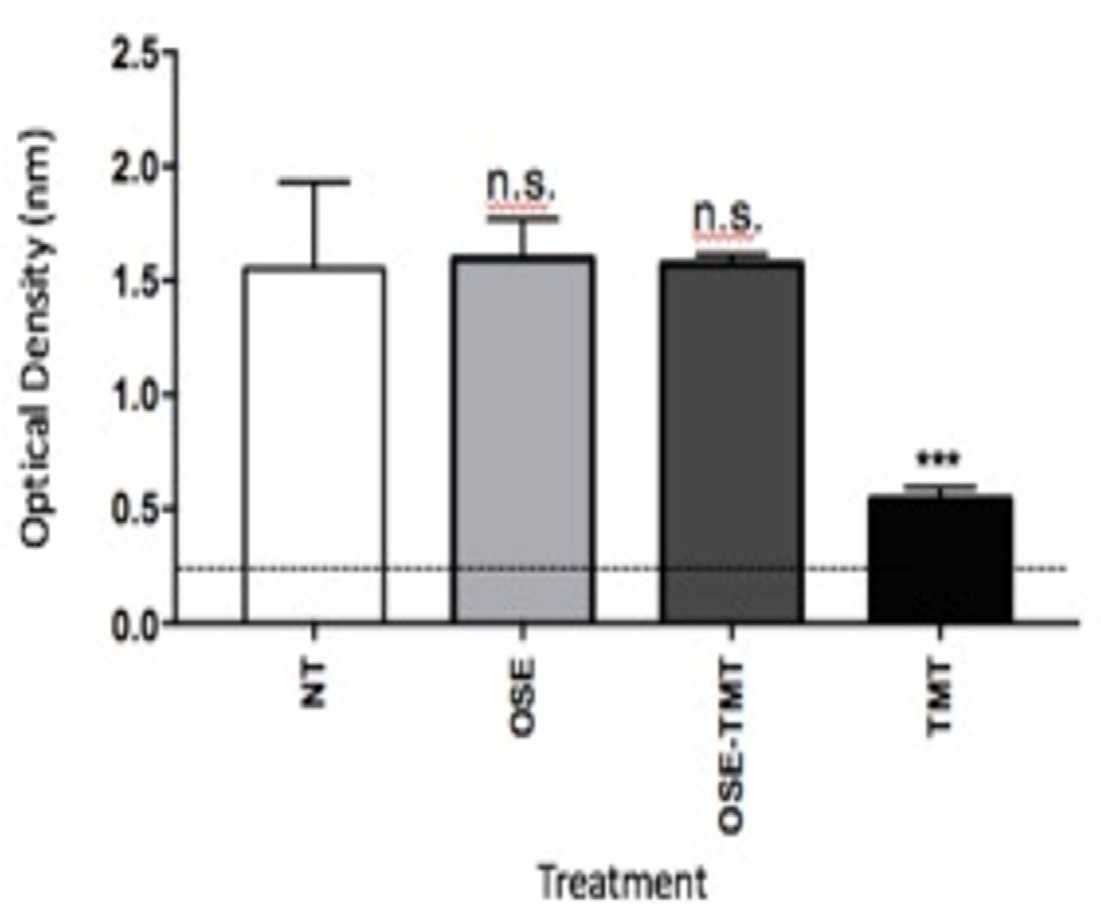
Serotonin (5-HT) activity on the HEK-293 cells line treated with TMT as in vitro model Alzheimer's disease (AD). Enzyme linked immunosorbent assay was performed to analysis the serotonin activity. Statistical analysis was performed by two-tailed Student's t-test. All experiment were performed in duplicate (n=3; NT= non treated cells; OSE = *Ocimum sanctum* Linn. ethanolic extract; TMT= Thrymethiltin Chloride; n.s.= non-significant; ***= significant)

The data below contain the raw analysis of the neuronal density on the CA1, CA3, DG hippocampal of AD rat model after treatment with the *Ocimum sanctum* ethanolic extract [1,2] (Fig. 1; Fig. 2; Fig 3; Table 1). Furthermore, in here we also performed the dataset of the serotonin expression [3,4] using immunohistochemistry on CA1, CA3, and DG of hippocampal in the AD rat model (Fig. 4; Fig. 5; Fig 6; Table 2; raw data in the Mendeley https://data.mendeley.com/datasets/4g74bxfvwr/1; DOI: 10.17632/4g74bxfvwr.1). Furthermore, there is also a visualization of the data of the serotonin expression by ELISA on the in-vitro model AD using Human embryonic kidney-293 (HEK-293) cells (Fig. 7). The HEK-293 is a cell line with the similar molecular pattern to the neuronal lineage cells. Several studies have shown a similarity between HEK-293 cells and neuronal cells in general, both in terms of morphology and protein expressed [2,5,6,7]

**Table 1:** The semiquantitative number of neuron density in CA1, CA3, and DG.

	CA1			CA3			DG		
	7 days	14 days	21 days	7 days	14 days	21 days	7 days	14 days	21 days
**A**	++++	++++	++++	++++	++++	++++	++++	++++	++++
**B**	++	++	++	++	++	++	++	++	++
**C**	+++	+++	+++	+++	+++	+++	+++	+++	+++

++++= a lot of +++=Numerous, ++= many +=A few, -=Not detected, CA1= Cornu Ammonis 1; CA3 = Cornu Ammonis 3; DG = Dentate Gyrus

**Table 2:** The semiquantitative number of serotonin expressions in CA1, CA3, and DG.

	CA1			CA3			DG		
	7 days	14 days	21 days	7 days	14 days	21 days	7 days	14 days	21 days
**A**	++++	++++	++++	++++	++++	++++	++++	++++	+++
**B**	+	-	-	++	-	-	++	-	-
**C**	+++	+++	+++	+++	+++	+++	+++	++	++

++++= a lot of +++=Numerous, ++= many +=A few, -=Not detected, CA1= Cornu Ammonis 1; CA3 = Cornu Ammonis 3; DG = Dentate Gyrus

## 2 Experimental Design, Materials, and Methods

### 2.1 Preparation of *Ocimum sanctum* Linn. ethanolic extract

*Ocimum sanctum* leaves verified by a botanist from the Department of Biology, Universitas Gadjah Mada, Yogyakarta, Indonesia then were prepared at The Central for Research and Development of Medicinal Plants and Traditional Medicines, Ministry of Health in Tawangmangu, Central Java, Indonesia. The extract was prepared as follows: 300 grams of the *Ocimum sanctum* Linn. powder macerated in four liter of 96% ethanol (Merck, Darmstadt, Germany). Maceration was repeated twice, followed by filtration. The resulting filtrate was concentrated using a vacuum rotary evaporator (Heidolph, Schwabach, Germany) under reduced pressure at 60°C. The final yield of the ethanolic extract *Ocimum sanctum* leaves was 8.82 % w/w.

## 3 Animal Model and Experimental Design

We used two months old 27 males rat (Wistar rats). The rats were derived from the Research Center of Biotechnology, Yogyakarta, Indonesia and had 250-300 g average of weights. Rats were maintained in individual cages with standard conditions (room temperature: 24–26°C; humidity: 60–65%) and free access to food and water 12/12 h daylight/dark. The rats consist of three groups (n=9) based on the type of treatment. Group A was given saline as a control. Then, group B was given pre-induced OSE 100 mg/kg BW for seven days orally followed by a single dose of TMT at 8 mg/kg BW intraperitoneally on the 7^th^ day. Group C pre-induced OSE was administered 100 mg/kg BW for seven days orally followed by a single dose of TMT at 8 mg/kg BW intraperitoneally and continued by OSE 100 mg/kg BB post-induction. Post-induced OSE will be given for 21 days. Perfusion was carried out on the 7th, 14th, and 21st days post-induction of TMT (three rat/group). After perfusion, brain samples were taken to make paraffin blocks for histological preparations, stained with cresyl violet and performed immunohistochemistry for serotonin (5-HT) receptors using serotonin antibody.

Cresyl violet staining provide an image of neuronal loss because of TMT toxicity. Immunohistochemistry of serotonin describe an image of neuronal activity during the action of OSE against TMT toxicity. Both of these data should help us understand the mechanism of OSE through neuronal loss and neuronal activities.

## 4 Brain Hippocampal Tissue Processing

Rats were anaesthetized using combination of 10% ketamine hydrochloride (Kepro, Maagdenburgstraat, The Netherland) and xylazine 2% (Interchemie, Metaalweg, The Netherland) intramuscularly. Rat placed dorsally and fixed. The thoracic cavum was opened with scissors until the heart appeared. Perfusion was began with the injection of pre-rinse liquid (0.9% NaCl with 0.1 g of NaCl at 100 ml of distilled water, 1 ml EDTA) via left ventricle and immediately followed by cut out of right atrium. After the pre-rinse fluid became clear, fixation using 4% formalin PBS solution applied intracardially. Brain removal done by reckless the head scalp until the skull arise. The brain then sequestered from its cranium by cutting off the cranial nerve and the olfactory bulb tip. Brain tissues were fixed in 4% formalin and processed with the paraffin-embedded method. Twenty micrometers thickness of tissue samples visualized by using Cresyl Violet staining and streptavidin-based immunohistochemistry method for 5-HT.

## 5 Cresyl Violet Staining

Nissl staining was conducted using cresyl violet (Sigma, Steinheim, Germany). The cresyl violet solution filtered with filter paper and incubate on the incubator at 37°C. On the following day the slides were put into an incubator at 37°C for 5 minutes and then washed with PBS (Thermofischer, Rockford, IL, USA) five minutes. Deparaffinization were performed using xylol 3 times in 5 minutes then followed by rehydration using graded series of alcohol in 3 minutes. The slides were arranged on a rack and stained with cresyl violet in an incubator for 30 minutes. The slides then rinsed with aquades in one minute, then dehydrated using Ethanol 70%, 80%, and 90%, Absolute Ethanol I and II, followed by Xylene I, II and III. Slide then mounting with balsam Canada and covered with cover glass. The formation of the hippocampal observed by using a light microscope (Nikon, Tokyo, Japan) at the magnification of 40 fold. Data analysis performed by microscopic readings software Optilab Image Viewer (Optilab, Yogyakarta, Indonesia). The amount of the cell in the hippocampal area (mm^2^) calculated by Optilab Image Raster software (Optilab, Yogyakarta, Indonesia).

## 6 Immunohistochemistry of Serotonin

Streptavidin-based immunohistochemistry method was performed to detect 5-HT on the hippocampal of rat model AD. Slides were deparaffinized followed by rehydrating and were rinsed in running water for 10 minutes. Distilled water placed in the microwave in 20 minutes for pre-heating antigen retrieval followed by placed slides into it, and incubated for 10 minutes on a low temperature microwave for antigen retrieval. Slide were washed in Phosphate Buffer Saline (PBS) 0.01 M (pH 7.4) for five minutes. The activity of endogenous peroxidase blocked by incubating the slides in 3% H_2_O_2_ in absolute methanol (1 mL H_2_O_2_ 30%, 9 mL absolute methanol) for 15 minutes at room temperature. The slides were washed with PBS (three times each 5 minutes) and dripped with Biocare Background Sniper (Biocare Medical, Prinsessegracht, Netherlands), incubated for 15 minutes in room temperature, placed horizontally in closed and moist box. Rabbit anti-5-HT (1:200) was used as a primary antibody (30 µl for each tissue section) and incubated for 48 hours at 4°C. Slides were washed with PBS three times for 5 minutes, respectively. The immunoreactivity cells were visualized using the Starr Trek Universal HRP Detection (Biocare Medical, Prinsessegracht, Netherlands). The DAB Substrate Kit ab94665 (Biocare Medical, Prinsessegracht, Netherlands) was treated as a chromogen. Slides were counterstained with Harris hematoxylene eosin (Leica, Wetzlar, Germany) and rinsed in running water for 10 min. Then, slides were dehydrated, cleared and mounted. The formation of 5-HT immunoreactivity cells were observed by using a light microscope (Nikon, Tokyo, Japan) at the magnification of 40 fold. Data analysis were done by microscopic readings software Optilab Image Viewer (Optilab, Yogyakarta, Indonesia). The amount of cells in the hippocampal area (mm^2^) calculated by Optilab Image Raster software (Optilab, Yogyakarta, Indonesia).

## 7 HEK-293 Preparation for ELISA

HEK-293 cells were grown on High Glucose DMEM media with 10% FBS supplementation, 1 % of antibiotic and 0.5% antifungal in a T75 flask then stored in a CO_2_ incubator. Cells (5 × 10^5^) were seeded on each well of 6 well plate, then were incubated for one hour. Cells were treated with OSE 50 ug/ml, 70 ug/ml, 100 ug/ml, 200 ug/ml, and nontreated cells run as a control then were incubated for 24 hours. The media were aspirated and the plate was washed using PBS one time. RIPA buffer (600 ul) was added on each well, the plate was shaken and rocked for 15 minutes. Cell scrappers were used to detach the cells for the bottom of the plate. The lysates were transferred to 1,5 ml microtubes. The plate was washed using 300 ul RIPA buffer then combined with the first lysate. The lysates were centrifuged at 10.000 xg speed for 10 minutes at 4 °C. The supernatants were transferred to new 1,5 ml microtubes and continued with the ELISA analysis.

## 8 ELISA for Serotonin

Competitive ELISA with 5-HT ELISA Kit (Fine Test, Wuhan, China) were performed. The plate was washed 2 times before adding standard, sample, and control (zero) wells. Standards and lysate samples (50 µl) were added into each followed by 50 µl Biotin-labeled antibody immediately then were incubated for 45 minutes at 37°C. The plate was aspirated and washed 3 times before added 100 µl HRP-Streptavidin Conjugate (SABC) into each well and incubated for 30 minutes at 37°C. Aspirated and washed 5 times then incubated with 90 µl TMB Substrate for 20 minutes at 37°C followed by 50 µlstop solution. Plate was read at 450 nm immediately, then the data were calculated.

## Appendix Supplementary materials

Image, application 1
